# Supplementary material for: CMV reactivation initiates long-term expansion and differentiation of the NK cell repertoire
Source: Front Immunol. 2022 Dec 1;13:935949. doi: 10.3389/fimmu.2022.935949 (PMC9753568; doi:10.3389/fimmu.2022.935949)
Supplement: Supplementary file 1 [file DataSheet_1.pdf]

Supplementary Figure 1. Gating strategy for NK cells from PBMCs.

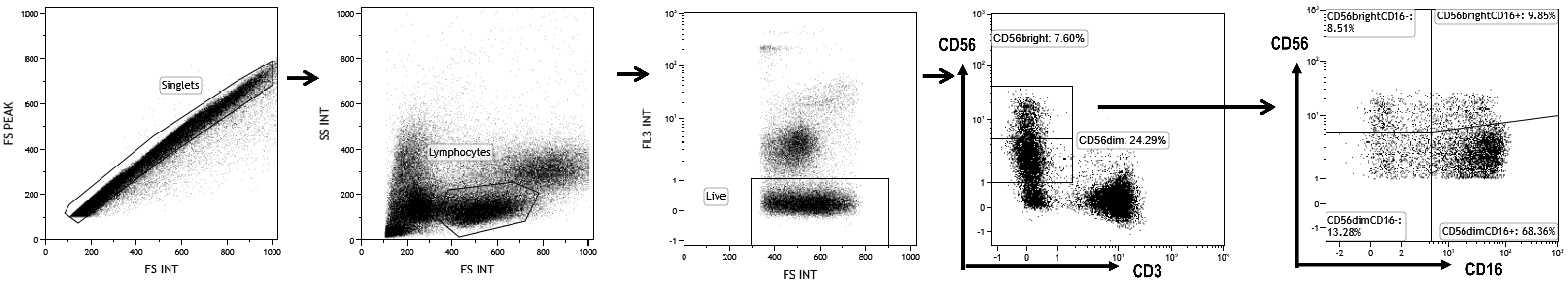

Figure S1. Gating strategies shown for the detection of 4 NK subsets population. At first the singles were gated, before the lymphocytes were gated. Then the whole NK cells were gated using CD3-CD56+ gate after the dead cell(PI+), B cells (CD19+) and monocytes (CD14+) were excluded. Then the 4 NK subsets were identified based on CD56 and CD16 expression from whole NK cells.

Supplementary Figure 2. Examples of NKG2C and NKG2A staining from four different NK subsets at 6 months post CMV reactivation after HSCT.

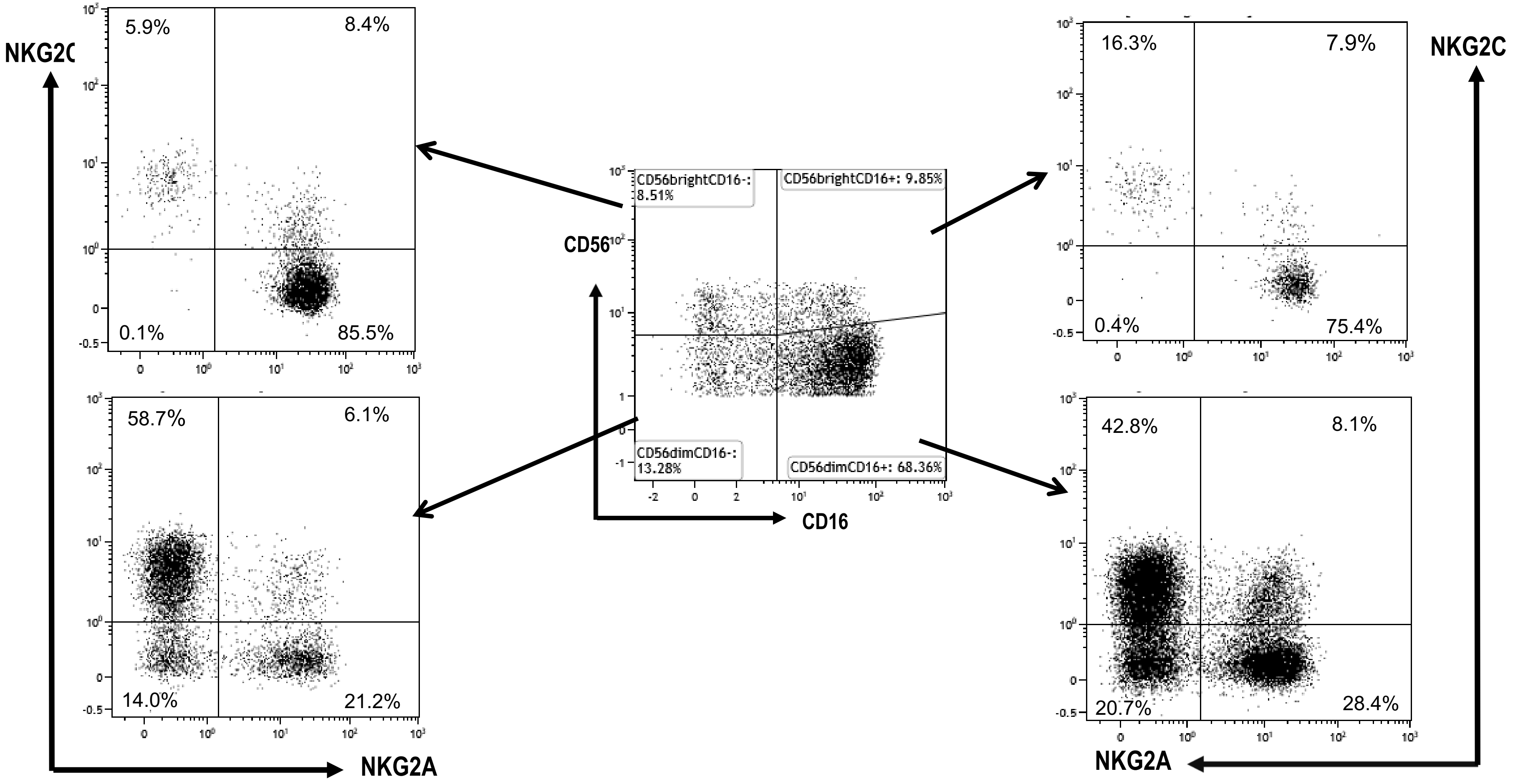

Figure S2. Example to show NKG2C/NKG2A expression pattern on 4 NK subsets, CD56^{brigh}CD16^{neg}, CD56^{bright}CD16^{bright}, CD56^{dim}CD16^{neg} , CD56^{dim}CD16^{bright} at 6 months post CMV reactivation from one HSCT patients.

Supplementary Figure 3. There is no significant difference for NK numbers and percentage between patients with single or multiple episode of CMV reactivation.

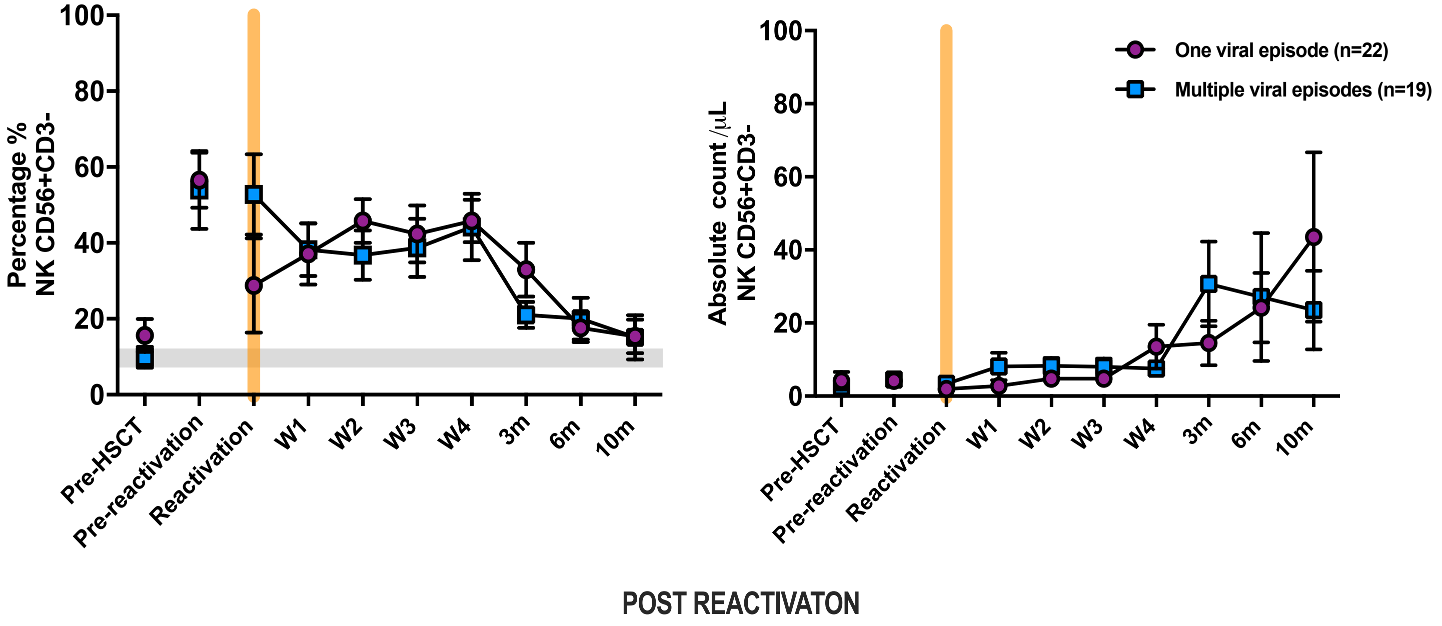

Figure S3. HSCT patients who underwent CMV reactivation were divided into two groups according to single or multiple episode of CMV reactivation. The NK cell percentage and absolute count were compared between these two group. Grey line is the NK cells percentage out of whole lymphocytes in Healthy Donors(HD). No data was available on absolute NK cell count in HD.

Supplementary Figure 4. Percentage of CD57, KIRs and CD69 positive NK cells following CMV reactivation in relation to donor serostatus.

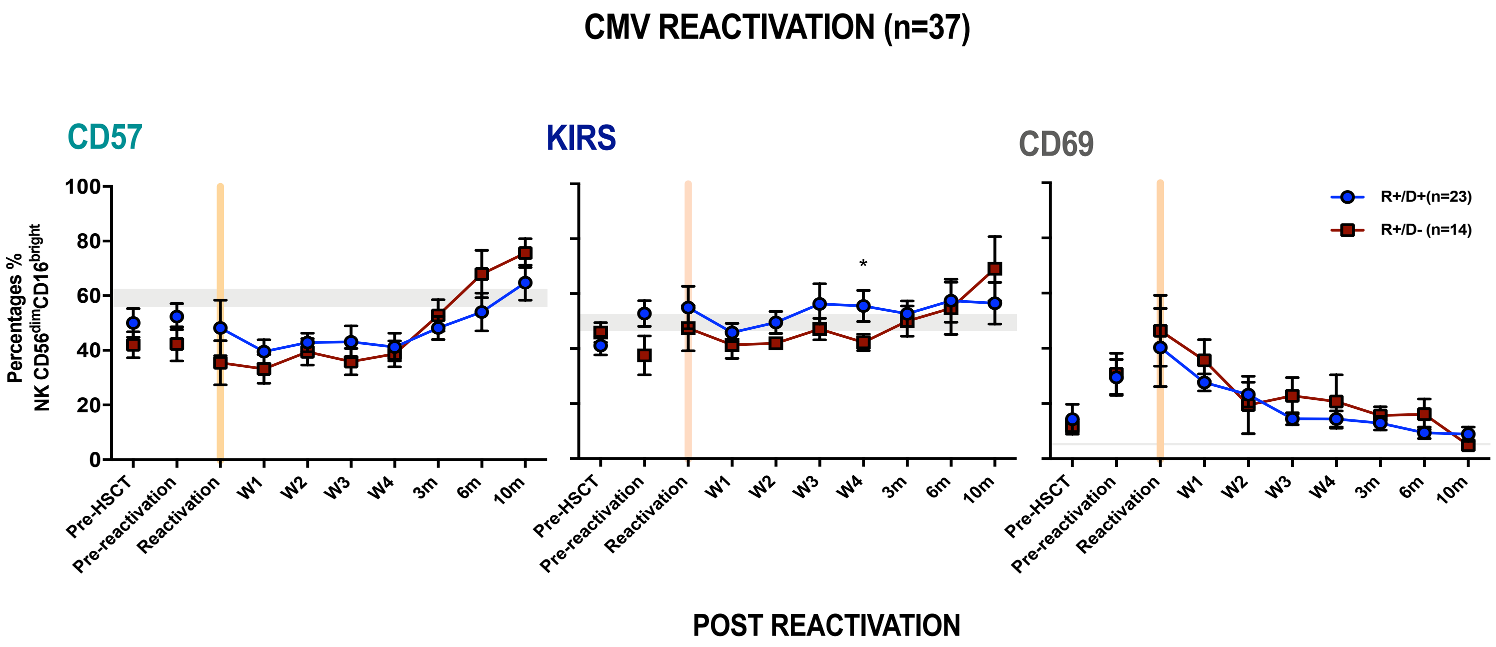

Figure S4. Percentage of CD57, KIRs and CD69 positive NK cells following CMV reactivation were compared between HSCT who underwent CMV reactivation according to donor CMV serostatus (R+/D+ in blue line and R+/D- in purple line). Grey lines represent the range of in healthy donors.

Supplementary Figure 5. NK cells from HSCT patients who have undergone CMV reactivation demonstrate higher KIRs expression.

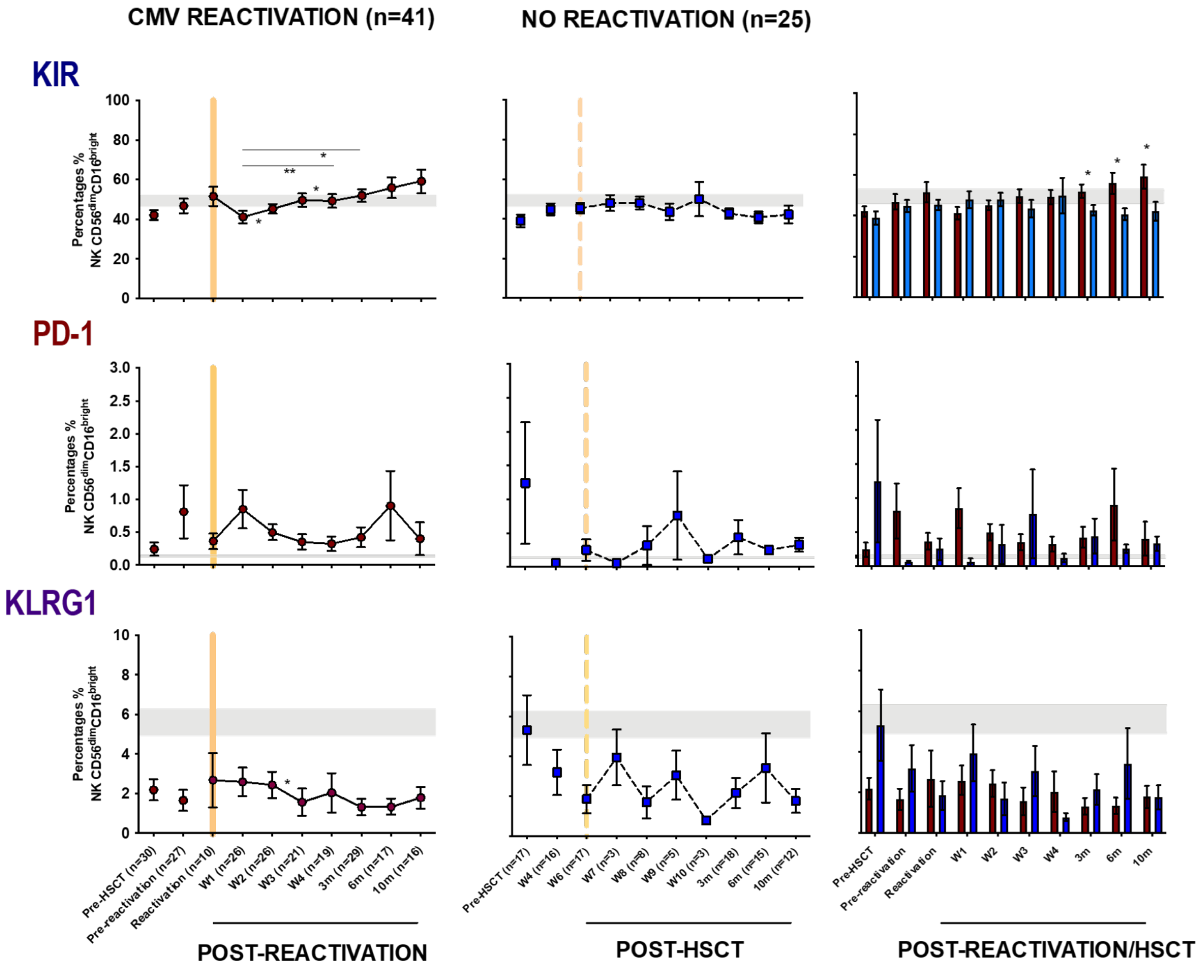

Figure S5. The percentage of KIRs, PD-1 and KLRG1 on NK cells from patients following CMV reactivation (purple line and bar) or without CMV reactivation (blue line and bar). Grey lines represent the range of NK cells in healthy controls. (*p<0.05, **p<0.01 Wilcoxon sign rank test for matched phenotypes)

Supplementary Figure 6. Percentage of CD69 positive NK cells following CMV reactivation in NK subsets according to NKG2A and NKG2C expression.

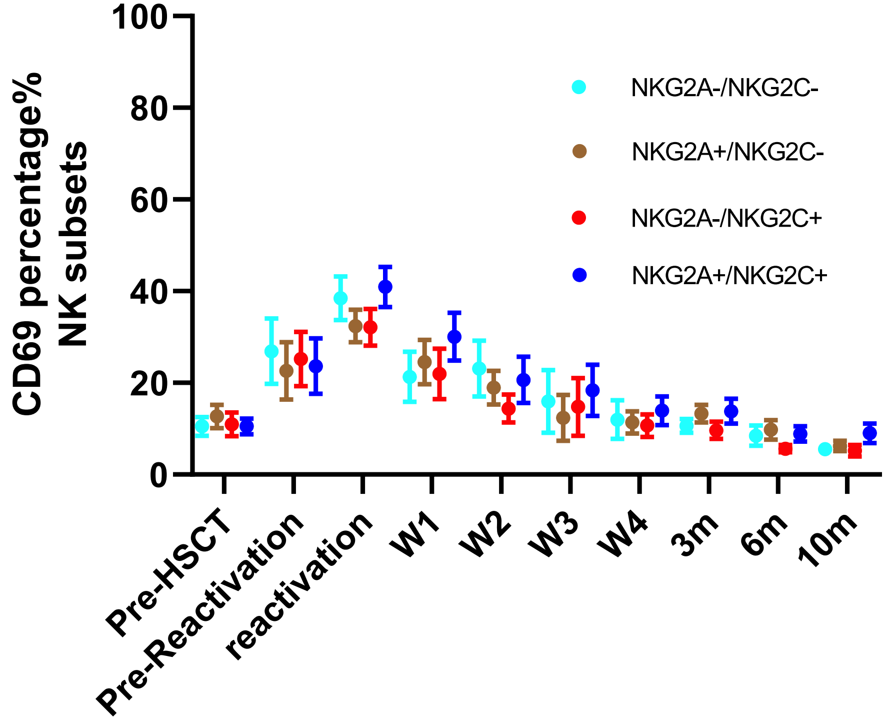

Figure S6. The percentage of CD69 expression on NK cells was studied over the time from pre-HSCT to 10 month after HSCT from patients with CMV reactivation. The CD69 expression was analysed from four different NK subsets: NKG2A-/NKG2C-, NKG2A+/NKG2C-, NKG2A-/NKG2C+, NKG2A+/NKG2C+. There is no statistically significant difference at all time points.

Supplementary Figure 7. Cytokines production following K562 stimulation in NK cells from HSCT patients with or without CMV reactivation between CD56^{dim} and CD56^{bri} NK subsets.

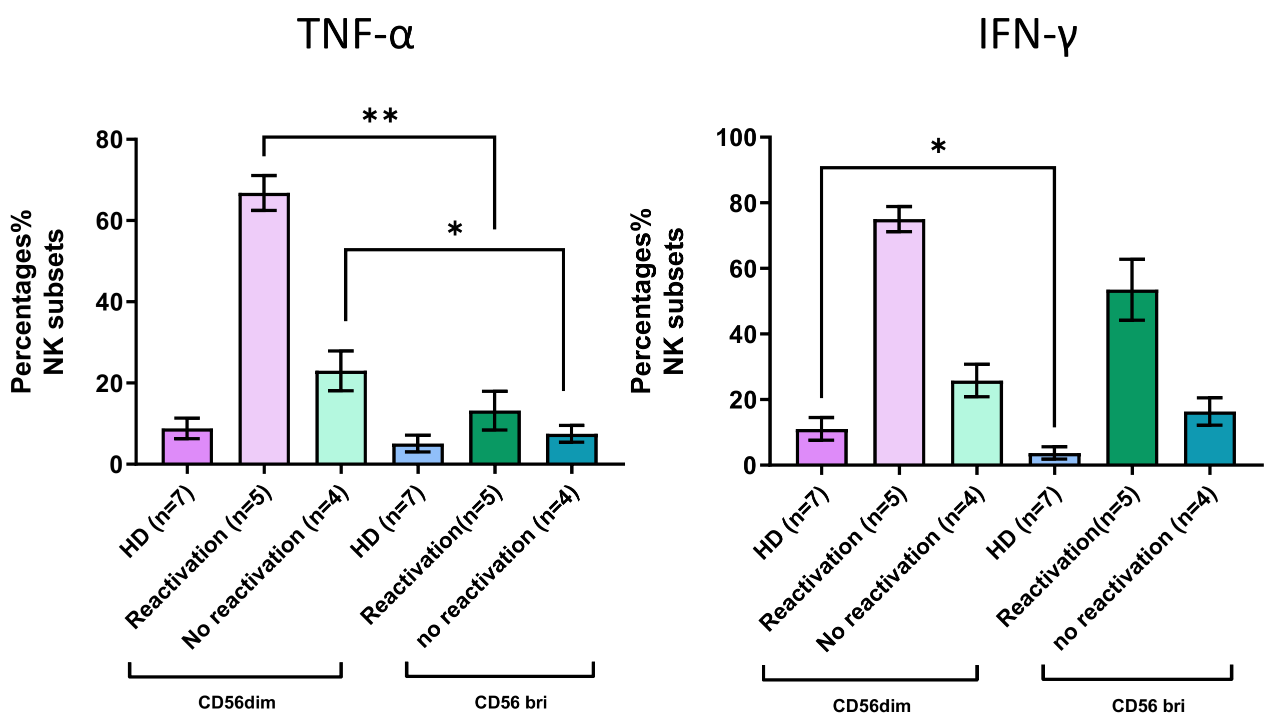

Figure S7. NK cells were stimulated with K562 overnight before the intracellular cytokines staining was carried out to study the IFN-γ and TNF-α production. The IFN-γ and TNF-α production from NK cells of HSCT patients was compared between the CD56^{dim} and CD56^{bri} NK subsets. Data are shown as percentage of cytokine-positive NK subsets following K562 stimulation. (*p<0.05**p<0.01; Anova test).
